# Epigenetic regulation of miR-129-2 and its effects on the proliferation and invasion in lung cancer cells

**DOI:** 10.1111/jcmm.12597

**Published:** 2015-06-17

**Authors:** Yingying Xiao, Xiaoxia Li, Haoli Wang, Ruiling Wen, Juan He, Jun Tang

**Affiliations:** KingMed Diagnostics and KingMed School of Laboratory Medicine, Guangzhou Medical UniversityGuangzhou, China

**Keywords:** microRNA, valosin-containing protein, DNA methylation, cell invasion, lung cancer

## Abstract

MicroRNAs (miRNAs) play a pivotal role in carcinogenesis. Dysregulation of miRNAs, both oncogenic miRNAs and tumour-suppressive miRNAs, is closely associated with cancer development and progression. The levels of miRNAs could be changed epigenetically by DNA methylation in the 5′ untranslated region (UTR) of pre-mature miRNAs. To investigate whether DNA methylation alters the expression of miR-129 in lung cancer, we did DNA methylation assays and found that 5′ UTR region of miR-129-2 gene was absolutely methylated in both A549 and SPCA-1 lung cancer cells, but totally un-methylated in 95-D cells. The expression of miR-129 was restored by 5-Aza-2’-deoxycytidine (DAC), a de-methylation agent, in both A549 and SPCA-1 cells, resulting in attenuated cell migration and invasion ability, and decreased protein level of NF-κB, which indicates the involvement of NF-κB pathway. To further illustrate the roles of miR-129 in lung tumourigenesis, we overexpressed miR-129 in lung cancer cells by transfection of miR-129 mimics, and found arrested cell proliferation at G2/M phase of cell cycle and inhibited cell invasion. These findings strongly suggest that miR-129 is a tumour suppressive miRNA, playing important roles in the development and progression of human lung cancer.

## Introduction

Lung cancer, including small-cell lung carcinoma (SCLC) and non-SCLC (NSCLC), is the leading cause of cancer-related mortality worldwide, with a 5-year survival rate of less than 15% 1–3.

microRNAs (miRNAs), a class of small non-coding RNAs in animals and plants, averaging 20 to 24 nucleotides, are known to bind to the 3′ untranslated regions (UTRs) of target mRNAs, resulting in mRNAs degradation or translational inhibition, through which miRNAs control a wide variety of physiological processes in normal cells, including proliferation, differentiation and apoptosis 4. Increasing attention has been paid to explicating the mechanism of miRNA regulation. As we know, the most of protein-coding genes may be regulated by epigenetics, one of which is DNA methylation frequently observed in the CpG islands sites on promoter region of a gene 5. It is said that 10% of miRNAs are undergoing epigenetic regulation through DNA methylation in the miRNA 5′ regulatory region 5,6.

The CpG islands are encompassed in the promoter and 5′ UTR region of miR-129-2 gene, located in chromosome 11p11.2. DNA methylation mediated downregulation of miR-129 has been reported in endometrial 7, gastric 8,9, colorectal 10, liver cancer 11, and lymphocytic leukaemia 12, and miR-129 expression level is much lower in tumour cell lines or primary tumour tissues from neural, gastric, and colorectal cancers than their corresponding controls 13–15. miR-129 is one of the candidate miRNAs possessing the potential of tumour suppressor activity, implied by studies that the ectopic expression of miR-129-5p reduced proliferation activities and promoted cell death of endometrial tumour cells and bladder cancer cells 7,16. In lung adenocarcinoma, it was reported that the expression level of miR-129 was much lower as compared with normal lung tissues 17. Here, we speculate that DNA methylation alters the expression of miR-129 in lung cancer and miR-129 plays pivotal roles in lung cancer progression. In the present study, we found that miR-129 was regulated by DNA methylation and conferred the tumour suppressive potential.

Valosin-containing protein (VCP), also known as p97, belongs to the AAA family (ATPase with multiple cellular activities) 18. The inhibition of VCP reduced NSCLC tumour growth in both *in vitro* and xenograft murine (athymic-nude) models after EerI treatment (*P* < 0.05) 19. In this study, we observed a reduction of VCP in either miR-129 overexpressing or hypomethylated lung cancer cells, and verified that VCP gene is a target of miR-129.

## Materials and methods

### Cell lines and cell culture

Human lung cancer cell lines 95-D, SPCA-1, A549, PC-9, SK-MES-1 and human embryonic kidney cells (HEK293) were purchased from Cell Bank at the China Academy of Science (Shanghai, China). All of the human lung cancer cell lines were propagated in RPMI medium 1640 (Life Technologies, Carlsbad, CA, USA) supplemented with 10% foetal bovine serum, 100 IU/ml penicillin and 100 μg/ml streptomycin (Life Technologies), while HEK 293 was grown in DMEM culture medium (Life Technologies) supplemented with 10% FBS, 100 IU/ml penicillin and 100 μg/ml streptomycin, and they were cultured at 37°C in a humidified 5% CO_2_ incubator. Cell transfections were done by using X-tremeGENE siRNA Transfection Reagent (Roche, Indianapolis, IN, USA) as the manufacturer described. For hypo-methylation, cells were treated by 2.5 μM DAC (Sigma-Aldrich, St. Louis, MO, USA).

### Genomic DNA isolation and bisulphite treatment

Total genomic DNA was isolated from cells by using DNAzol (Life Technologies) according to the manufacturer’s instructions. Bisulphite treatment of DNA was carried out with the EpiTect Bisulfite kit (Qiagen, Valencia, CA, USA) following the manufacturer’s instructions.

### CpG Island methylation detection by touch-down (TD) methylation-specific polymerase chain reaction

DNA sequences of miR-129-2 promoter and 5′ UTR region were analysed with the MethPrimer program 20 to design methylation-specific and unmethylation-specific primers for methylation-specific polymerase chain reaction (MSP) as listed in Table S1. Amplification reactions were carried out on the Bio-Rad CFX96 RealTime PCR System (Bio-Rad, Hercules, CA, USA). The TD-PCR program was shown in Table S2. Each run included non-template control (using sterile water as template), an unmethylated control and a methylated control (Qiagen). All the experiments were repeated for at least three times.

### RNA isolation and real-time RT-PCR

Total RNA was extracted using a standard TRIzol (Life Technologies) protocol. For miRNA real-time RT-PCR, All-in-One miRNA qRT-PCR Detection Kit (GeneCopoeia, Rockville, MD, USA) was used according to the manufacturer’s instructions, and specific forward primers of miRNAs were synthesized by GenePharma (Shanghai, China). For mRNA real-time RT-PCR, SuperScript III one-step RT-PCR kit (Life Technologies) was used according to the manufacturer’s instructions. Specific primers for mRNA detection are shown in Table S1. The expression of mRNA was determined using the 2^−ΔΔCT^ method. All the experiments were repeated for at least three times.

### Protein extraction and immunoblotting

Western blot analysis was performed as described previously 21. The blots were photographed by the ChemiDoc XRS gel documentation system (Bio-Rad). The assay was performed in three independent experiments.

### Luciferase assay

The different portions for 3′-UTR of VCP gene covering the two binding sites predicted were amplified by PCR using specific primers (Table S1) for wild type (WT), for the deletion of the first binding site (DEL1), for the deletion of the second binding site (DEL2), and for the deletion of both of the binding sites (DEL3). The PCR products were subcloned to the downstream of the *Renilla* luciferase (Rluc) gene in a modified psiCHECK-2 vector (psiCHECK-2 (M)), as described by Zhou *et al*. 22. HEK293 cells grown on 96-well plates were co-transfected with both psiCHECK-2 (M) construct and miR-129 mimics or control. After transfection for 48 hrs, the cells were lysed and subjected to luciferase assay using the dual luciferase assay (Promega, Madison, WI, USA). Each transfection was repeated in triplicate.

### Cell proliferation assay

The MTT assay was performed as previously described 23. This assay was repeated at least three times.

### Trypan blue excluding assay

The assay was done as previously described 23.

### Wound healing assay

Cells were seeded into 6-well plate and cultured overnight before transfection with miR-129 mimics or inhibitor, and before DAC treatment. After 48 hrs, the cell monolayers were gently scratched with a pipette tip across the diameter of the well and rinsed with media to remove cellular debris. The surface distance of the scratch was quantified immediately after wounding and then measured again 24 hrs later through a microscope. This assay was repeated at least three times.

### Transwell assays for cell migration and invasion

A total of 1 × 10^5^ cells for each well were resuspended in 100 μl RPMI medium 1640, and added to the top chambers with 8-μm pore sized filter inserts (Corning Costar, Tewksbury, MA, USA). For cell invasion assay, before seeding cells, the top surface of the filter membrane was coated with 30% of BD Matrigel matrix (BD Biosciences, San Jose, CA, USA). For cell migration assay, Matrigel coating was not performed. The bottom chambers were soaked by complete medium. After overnight incubation, cells on the top surface of the filter membranes were removed, then the migrated or invaded cells were fixed by methanol and stained with crystal violet before counted. Experiments were repeated a minimum of three times.

### Flow cytometry

Cell sample preparation and staining were done as previously described 23. All the samples were analysed by FACSCalibur using CellQuest software (BD Biosciences).

### Statistical analysis

All statistical analyses were performed using Student’s *t-test*. All experimental data are presented as mean ± SD. *P* < 0.05 was considered significant, and the level of significance was assigned as * if *P* < 0.05 and as ** if *P* ≤ 0.01, respectively.

## Results

### Profiles of miR-129-2 gene methylation in lung cancer cell lines

We first predicted the CpG islands in DNA sequences of promoter and 5′ UTR region of miR-129-2 gene located in chromosome 11p11.2 by using the MethPrimer, as shown in Figure S1A. We performed MSP combining with technique of TD-PCR on five lung cancer cell lines and the location of MSP products was indicated in Figure S1A. We found that there was a clear band of 188 bp in 95-D cells amplified with un-methylated primers, whereas no band was found with methylated primers (Fig. S1B). For A549, PC-9, SPCA-1 and SK-MES-1, there was no band found with un-methylated primers; however, obvious bands of 188 bp were detected using methylated primers (Fig. S1B). In the subsequent experiments, A549 and SPCA-1 were chosen as the totally methylated model for miR-129-2, while 95-D as un-methylated model.

### VCP gene is a target of miR-129 and the hypomethylation treatment down-regulated VCP expression in lung cancer cells

**Figure 1:**
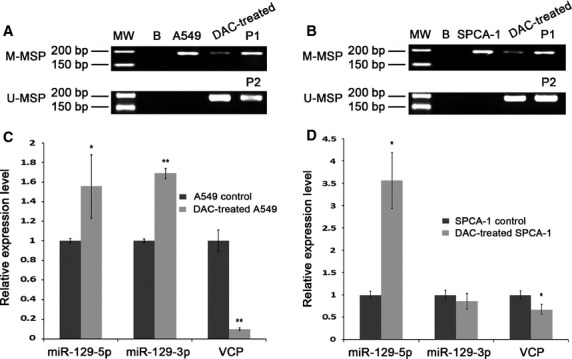
Effects of hypomethylation treatment on A549 and SPCA-1 cells by DAC. (A and B) Electrophoresis of MSP assay products for detection of miR-129-2 methylation after DAC treatment of A549 and SPCA-1 cells, respectively. The upper panel showed the results obtained using the methylated primers, while the lower panel indicated data with the un-methylated primers. MW: molecular weight marker; B: background control in which water was used as primer for PCR; P1: methylated control; P2: unmethylated control. (C and D) Real-time RT-PCR detection of miR-129-5p, miR-129-3p and *VCP* after DAC treatment of A549 and SPCA-1 cells, respectively.

**Figure 2:**
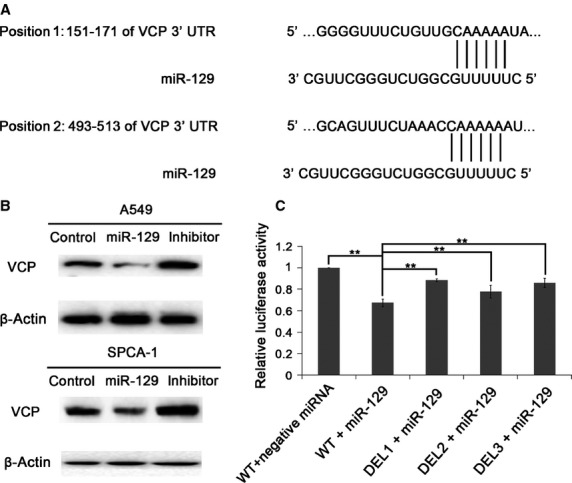
*VCP* mRNA is a direct target of miR-129. (A) Two putative miR-129-binding sites exist in the 3′-UTR of VCP gene. (B) VCP protein level was determined in A549 (upper panel) and SPCA-1 (lower panel) cells with overexpressed or down-regulated miR-129. (C) Co-transfection of miR-129 and psiCHECK-2 (M) -WT (wild type of 3′-UTR of VCP gene) inhibited firefly luciferase activity compared with control, and the firefly luciferase activities were increased in the cells transfected by plasmids with deletions of miR-129-binding sites, such as psiCHECK-2 (M) -DEL1 (deletion of position 1 in A), -DEL2 (deletion of position 2 in A) and -DEL3 (deletion of both position 1 and 2 in A), as compared with the one transfected with psiCHECK-2 (M) -WT.

To investigate the effect of hypomethylation on miR-129-2 totally methylated cells such as A549 and SPCA-1, 2.5 μM DAC, a demethylating agent, was added to the cell culture medium. As indicated in Figure1A, A549 cells showed faint band representing methylated miR-129-2 and strong band for un-methylated miR-129-2 after hypomethylation. The same results were observed in SPCA-1 cells (Fig.1B). To assess the alteration of miR-129 expression caused by hypomethylation, we performed real-time RT-PCR, and found that both of its mature products (miR-129-5p and miR-129-3p) were significantly increased in A549 cells (Fig.1C); while in SPCA-1, miR-129-5p rose predominantly and miR-129-3p did not change (Fig.1D). These data indicated that miR-129 was restored by DAC in both A549 and SPCA-1 cells. At the same time, we checked VCP gene, a presumed target gene of miR-129, by real-time RT-PCR, and discovered a marked decrease at *VCP* mRNA level in both cells (Fig.1C and D). To confirm the regulation role of miR-129 on *VCP*, we utilized luciferase assay for detection of the interactions between miR-129 and the putative miR-129-binding sites on 3′-UTR of VCP gene (Fig.2A), predicted by software TargetScanHuman. After transfection by miR-129 mimics or inhibitor, both of A549 (Fig.2B, upper panel) and SPCA-1 (Fig.2B, lower panel) cells showed weaker band or stronger band of VCP protein, respectively, by Western blotting. The results of luciferase assay showed significant inhibition of luciferase activity after miR-129 transfection (Fig.2C). After deletion of one or both of the two putative miR-129-binding sites, the luciferase activities rose remarkably (Fig.2C), suggesting that miR-129 directly regulates VCP gene through these two binding sites.

### Inhibition of the migration and invasion of hypomethylated A549 and SPCA-1 cells

**Figure 3:**
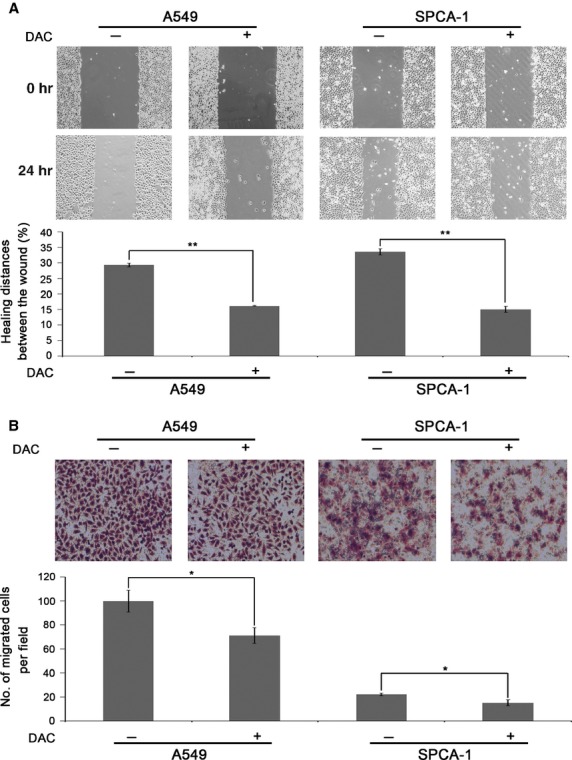
Inhibition of the migration of A549 and SPCA-1 cells by hypomethylation treatment. (A) The influence of hypomethylation treatment on lung cancer cell migration was determined by wound healing assay in A549 and SPCA-1 cells treated with DAC. Cells were seeded into 6-well plate and treated with 2.5 μM DAC for 2 days prior to starvation by using serum-free medium, and then subjected to wound healing assay. (B) Inhibition of cell migration by Transwell assay in A549 and SPCA-1 cells treated with DAC, respectively. Cells were seeded into 12-well plate and treated with 2.5 μM DAC for 2 days and then processed to Transwell assay for cell migration.

**Figure 4:**
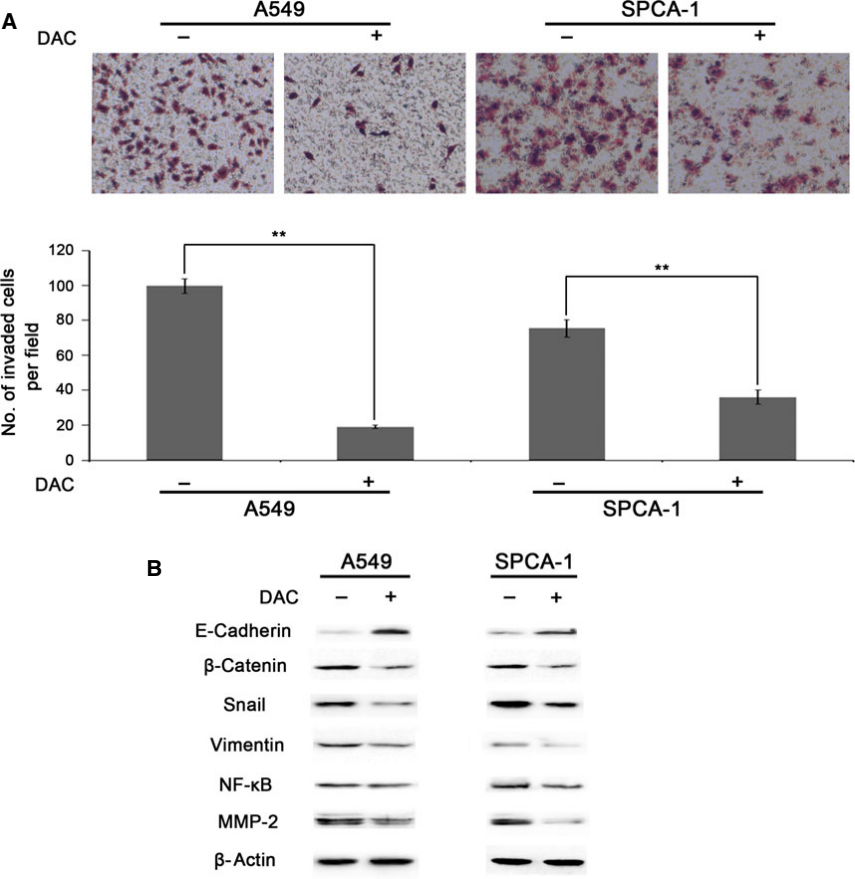
Inhibition of the invasion of A549 and SPCA-1 cells by hypomethylation treatment. (A) Hypomethylation inhibition of cell invasion was detected by Matrigel Transwell assay in A549 and SPCA-1 cells treated with DAC, respectively. Cells were seeded into 12-well plate and treated with 2.5 μM DAC for 2 days and then processed to Matrigel Transwell assay for cell invasion. (B) Detection of EMT related molecules by Western blotting in A549 and SPCA-1 cells treated with DAC, respectively. Cells were seeded into 6-well plate and treated with 2.5 μM DAC for 3 days and then processed to Western blot assay for specific protein determination.

We next examined the influences of hypomethylation on cell proliferation and viability, and no influence was found between before and after DAC treatment (Fig. S2A–D). We then employed wound healing assay and Transwell assay for detection of cell migration and invasion. After DAC treatment, A549 cell wound closure was 13.12% less than control cells (Fig.3A), whereas hypomethylated SPCA-1 cells migrated 18.42% less of wound closure compared to control (Fig.3A). Figure3B showed representative photographs of Transwell assay for cell migration, and the data showed 28.76% and 31.82% less migrated cell numbers in A549 and SPCA-1, respectively, after DAC incubation. We next investigated the effects of DAC on cell invasion by Matrigel Transwell assay. As a result, a striking difference was found of 80.94% and 52.21% less cells per field in DAC-treated A549 and SPCA-1 cells, respectively, compared to controls (Fig.4A). And we performed Western blotting on epithelial-mesenchymal transition (EMT) related proteins. The results showed a notably elevated protein level of E-cadherin, an active suppressor of invasion for many epithelial cancers, as compared with control cells (Fig.4B). Conversely, the expression levels of β-catenin, Snail and Vimentin were reduced (Fig.4B). We further examined NF-κB signal pathway which contributes to cell metastasis, and found that bands for NF-κB and its down-stream effector MMP-2 were much fainter after DAC treatment compared with control cells (Fig.4B). Taken together, these results showed that hypomethylation by DAC in lung cancer cells not only inhibited cell migration, but also inhibited cell invasion through down-regulation of β-catenin, Snail and Vimentin, as well as up-regulation of E-cadherin, involving the inhibition of NF-κB and MMP-2 expression.

### Suppression of cell proliferation with G2/M phase cell cycle arrest in miR-129 overexpressing A549 cells

**Figure 5:**
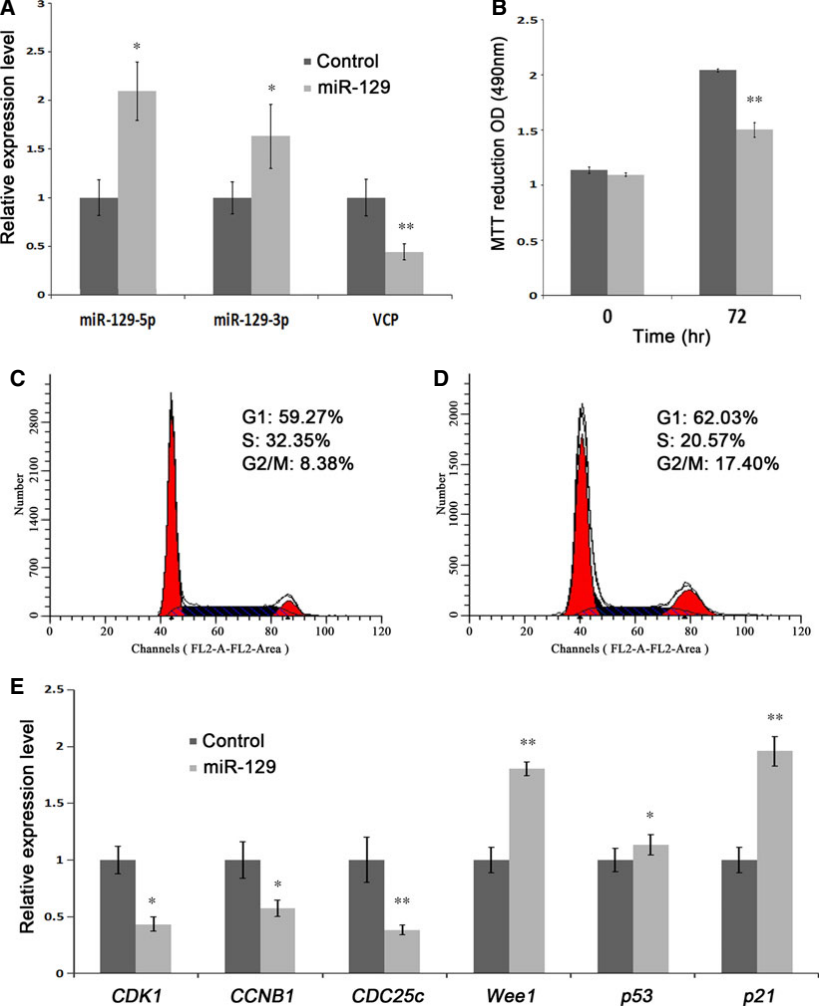
Effects of miR-129 overexpression on A549 cell proliferation and cell cycle. (A) Expression levels of miR-129-5p, miR-129-3p and VCP were determined by real-time RT-PCR in miR-129 overexpressing A549 cells. Cells were transfected with miR-129 mimics or the control for miR-129 mimics, and collected after 48 hrs incubation for real-time RT-PCR assays. (B) Inhibition of cell proliferation in miR-129 overexpressing A549 cells. Cells were seeded into 96-well plate and transfected with miR-129 mimics or the control for miR-129 mimics, and assessed by MTT assay for cell proliferation at 0 and 72 hrs after transfection. (C and D) miR-129 overexpression induced cell cycle arrest. A549 cells were transfected with miR-129 mimics (D) or the control for miR-129 mimics (C), and 48 hrs later, the cells were stained by PI and assessed for cell cycle distribution by flow cytometry. (E) Profiles of molecules related to G2/M processing determined by real-time RT-PCR. A549 cells transfected with miR-129 mimics or the control for miR-129 mimics were collected after 48 hrs, and examined for the expression levels of selected genes using specific primers.

To investigate the cellular roles of miR-129 in lung cancer cells, we conducted a functional knock-in study in a lung cancer cell line A549, which harbours epigenetically silenced miR-129. By real-time PCR, the overexpression of miR-129 increased the expression of miR-129-5p and miR-129-3p by 2.10-fold and 1.63-fold, respectively, and reduced *VCP* mRNA by more than 50% (Fig.5A) compared to the controls. Figure5B showed that the proliferation was reduced approximately 30% in miR-129 overexpressing cells compared to controls, as measured by MTT assay, indicating that the knock-in of miR-129-5p profoundly reduced the proliferation of A549 cells. Furthermore, we found that A549 cells were arrested at G2/M phase of cell cycle by miR-129 overexpression (Fig.5C and D). To delineate regulators for this observation, we ran real-time RT-PCR and found that the mRNA levels of *CDK1* and *CCNB1,* critical determinants of G2/M progression, as well as *CDC25c*, which could activate CDK1, reduced significantly compared with control cells (Fig.5E). However, *Wee1*, supposed to inactivate CDK1 by phosphorylations at Thr14 and Tyr15, increased by 1.80-fold at mRNA level (Fig.5E). Other negative regulators of G2/M processing such as *p53* and *p21* were dramatically up-regulated compared to control cells (Fig.5E). These data showed that miR-129 overexpression suppressed cell proliferation with G2/M phase cell cycle arrest in A549 cells through up-regulating *Wee1* and *p21* along with down-regulating *CDK1, CCNB1* and *CDC25c*.

### miR-129-2 overexpression inhibited cell migration and invasion in A549 cells

**Figure 6:**
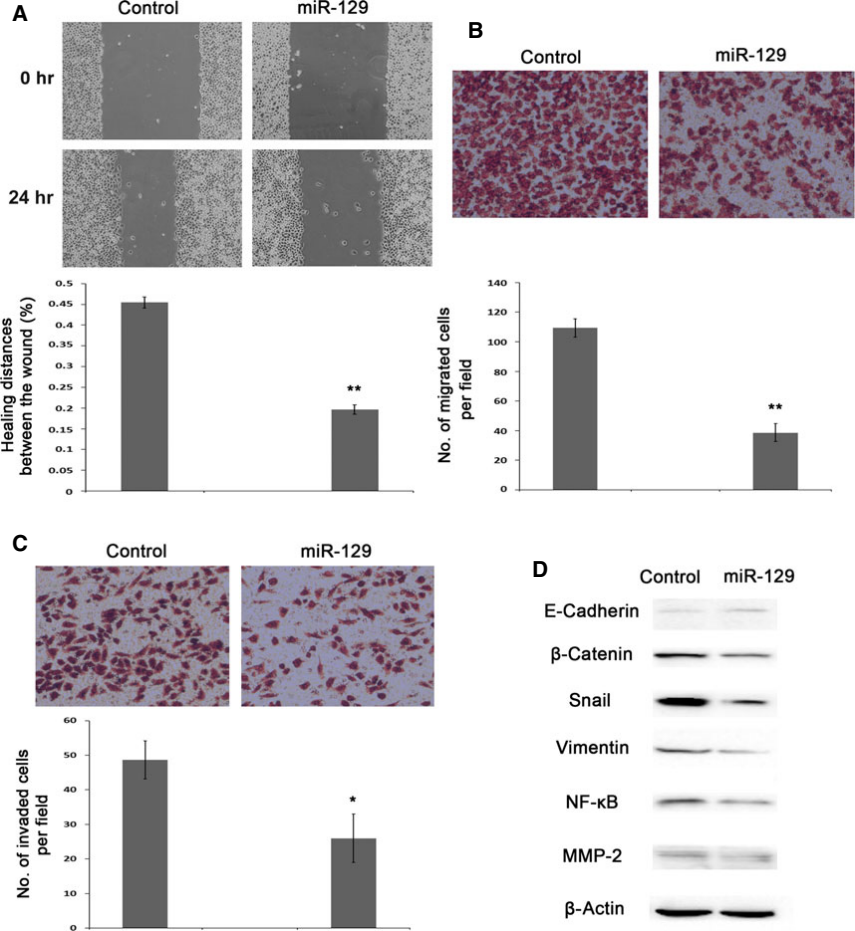
Inhibition of A549 cell migration and invasion by miR-129 overexpression. (A) Determination of cell migration ability by wound healing assay on A549 after miR-129 overexpression. Cells were seeded into 6-well plate and transfected with miR-129 mimics or the control for miR-129 mimics, incubated for 2 days prior to starvation by serum-free medium, and then subjected to wound healing assay. (B) Influece of miR-129 overexpression on cell migration determined by Transwell assay. (C) Influece of miR-129 overexpression on cell invasion assessed by Matrigel Transwell assay. Cells were seeded into 12-well plate and transfected with miR-129 mimics or the control for miR-129 mimics, and then processed to Transwell assays without or with Matrigel for cell migration or invasion after 48 hrs transfection. (D) Profiles of EMT related proteins by Western blotting in miR-129 overexpressing A549 cells. Cells were collected for experiments 48 hrs after transfection.

To study the effects of miR-129 on cell migration and invasion in lung cancer cells, we employed wound healing assay and Transwell assay. Figure6A exhibited representative photographs of wound healing assays, and the percentage of wound healing distance was 43.20% less in miR-129 overexpressed A549 cells than the control (Fig.6A, lower panel). Consistently, the number of migrated cells per field was 2.83-fold less in miR-129 overexpressed cells compared with the control utilizing Transwell assay (Fig.6B). The *in vitro* invasion of A549 cells was measured by a Matrigel Transwell assay, and miR-129 overexpression caused a 1.87-fold decrease in the number of invaded cells per field (Fig.6C). To sum up, these observations indicated that the up-regulation of miR-129 in lung cancer cells resulted in the inhibition of cell migration and invasion. We further evaluated the molecules involved in EMT process by Western blotting, and found a stronger band of E-Cadherin and weaker bands of β-Catenin, Snail and Vimentin compared with controls (Fig.6D). The NF-κB pathway was attenuated by miR-129 overexpression as indicated by fainter band than controls (Fig.6D). And MMP-2, a protein regulated by NF-κB pathway, presented much weaker band compared to that of controls (Fig.6D).

## Discussion

In this study, we examined the epigenetic status of miR-129-2 in five human lung cancer cell lines and found that miR-129-2 was absolutely methylated in A549, SPCA-1, SK-MES-1 and PC-9 cells, and totally un-methylated in 95-D cell, suggesting a methylation of miR-129-2 in the majority of lung carcinoma.

To investigate the roles of miR-129 in lung cancer, we determined the functions of miR-129 by ectopic expression in A549 cells. Our data showed that lung cancer cells were profoundly arrested at G2/M phase of cell cycle after miR-129 overexpression. The major regulators of G2 to M transition are CDK1 and the regulatory subunit cyclin B1 3, both of which showed reduced expression in miR-129 overexpressed cells. Wee1 could phosphorylate CDK1 at Thr14/Tyr15, resulting in inactivation of CDK1; while CDC25c could activate CDK1 by de-phosphorylation of Thr14/Tyr15 and induces formation of the CDK1/cyclin B1 complex, which in turn leads to M-phase transition of cell cycle 24,25. Our results demonstrated reduced *CDC25c* and increased *Wee1*, *p21,* and *p53*. It has been shown that the G2/M arrest is associated with increased level of the cyclin-dependent kinase inhibitor p21/WAF1 26 and p53. Studies revealed that p21/WAF1 blocked the initiation of early mitotic events by binding to cyclin B1 and CDK1 and sequestered them in the nucleus 27. The tumour suppressor p53 has been shown to arrest cells at G1-phase 28, also has the potential of hindering G2/M progression 29,30.

We also evaluated roles of miR-129 in lung cancer cell migration and invasion. Interestingly, in line with the observations after DAC treatment, the ectopic expression of miR-129 in A549 cells not only prevented the cell migration, but also hampered the cell invasion. Epithelial-mesenchymal transition endows cancer cells the properties of migration and invasion by activation of a variety of signalling pathways, including NF-κB pathways and their target genes 31–34. During EMT, the molecular repertoire of a cell experiences dramatic changes. We observed an increase in E-Cadherin, and a decrease in β-catenin, Snail, Vimentin and NF-κB in miR-129 ectopic expressed cells as compared with control, which were coincident with the cells treated with DAC. The decline of NF-κB, a critical metastatic regulator, caused apparently reduced protein level of MMP-2 which is responsible for the degration of basement membranes and stromal extracellular matrix and is of pivotal importance in tumour metastasis. The promoter of MMP-2 is highly conserved and shown to contain multiple functional elements, including NF-κB and AP-1 elements 35,36.

Moreover, we confirmed that VCP gene is a target of miR-129, and expressed negatively correlated with miR-129. Valosin-containing protein can interact with more than 30 different cellular proteins and is associated with ubiquitin/protesome-dependent protein degradation, endoplasmic reticulum (ER)-associated degradation, membrane fusion, transcriptional regulation, programmed cell death and so on 37–40. In hepatocellular and gastric carcinoma, the elevated expression of VCP is combined with increased incidence of recurrence, whereas the level of VCP in hepatocellular carcinoma tissues was negatively associated with the level of miR-129-5p 41–44. Whether VCP plays key roles in the cellular functions of miR-129 in lung cancer still needs to be further investigated.

In summary, miR-129 suppressed lung cancer cell proliferation by arresting cell cycle at G2/M phase through inactivation of CDK1 by Wee1, and reduced cell migration and invasion in lung cancer through controlling the protein levels of NF-κB and MMP-2.

## Supporting Information

**Figure S1** Profiles of miR-129-2 gene methylation in NSCLC cell lines.

**Figure S2** Influence of hypomethylation on cell proliferation of A549 and SPCA-1 cells.

**Table S1** Primers used in this study. **Table S2** The programs of TD-PCR.
