# Supplementary figures and images for: Epigenetic regulation of miR-129-2 and its effects on the proliferation and invasion in lung cancer cells
Source: J Cell Mol Med. 2015 Jun 17;19(9):2172–80. doi: 10.1111/jcmm.12597 (PMC4568922; doi:10.1111/jcmm.12597)

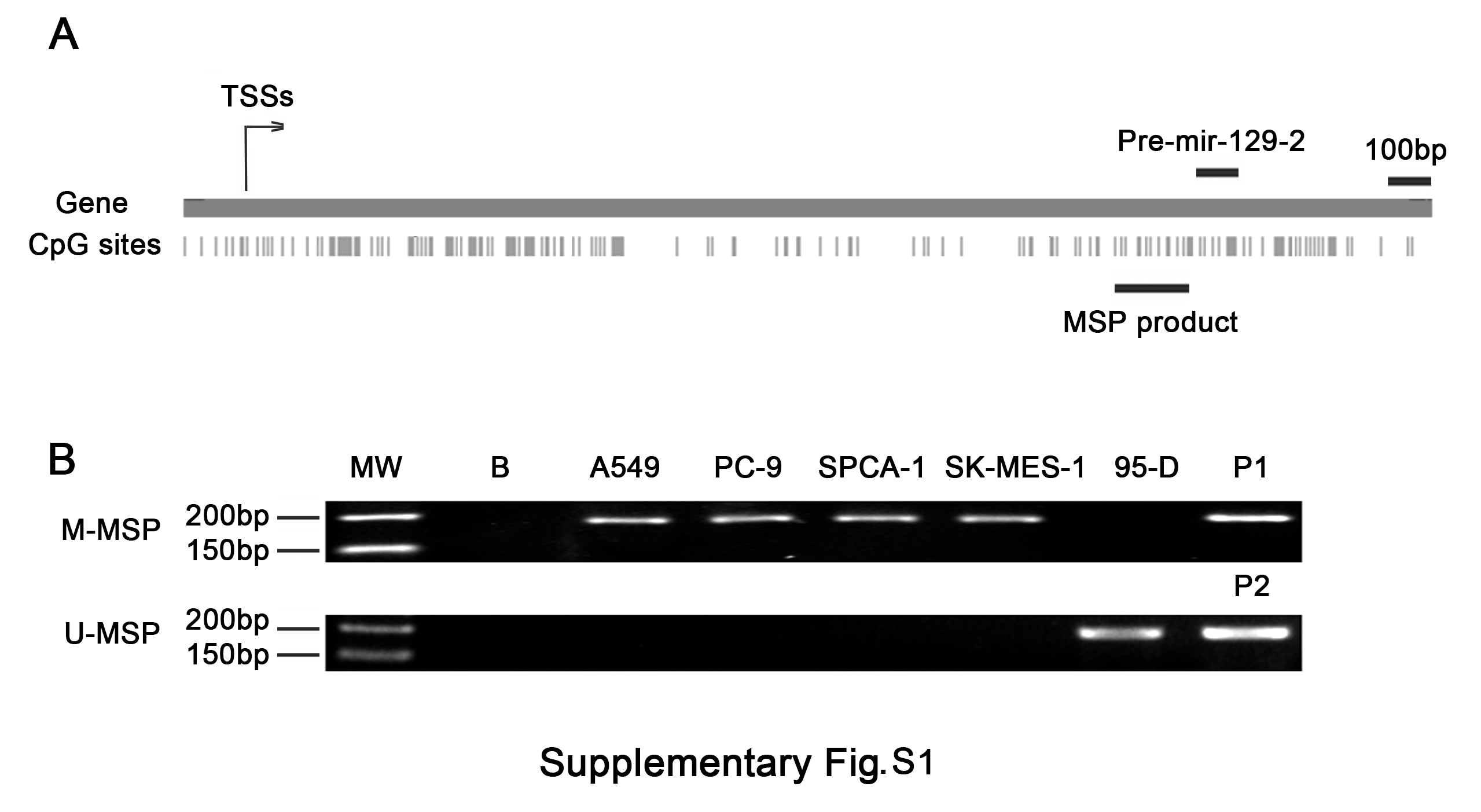

Supplement: Supplementary file 1 [file jcmm0019-2172-sd1.tif]

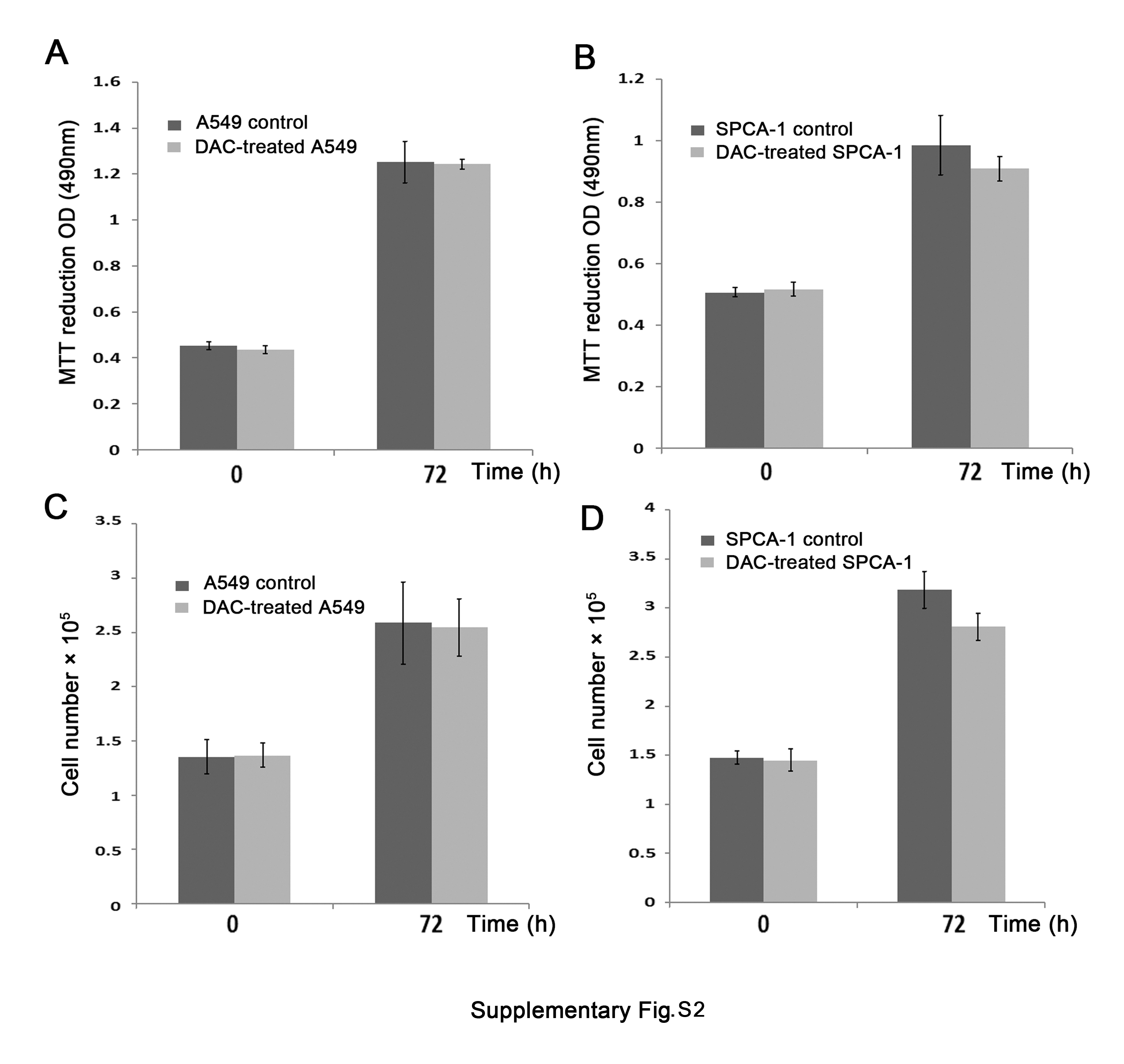

Supplement: Supplementary file 2 [file jcmm0019-2172-sd2.tif]
