# Supplementary material for: Epigenetic regulation of miR-129-2 and its effects on the proliferation and invasion in lung cancer cells
Source: J Cell Mol Med. 2015 Jun 17;19(9):2172–80. doi: 10.1111/jcmm.12597 (PMC4568922; doi:10.1111/jcmm.12597)
Supplement: Supplementary file 3 [file jcmm0019-2172-sd3.docx]

**Tables**

**Supplementary Table 1.** Primers used in this study

|  |  |
| --- | --- |
| **Primer sequence (5' to 3')** | **Description of experiment** |
| GAGTTGGGGGATCGCGGAC | Forward methylation specific primer for bisulfite PCR of the 5’ UTR region of miR-129-2 gene. |
| ATATACCGACTTCTTCGATTCGCCG | Reverse methylation specific primer for bisulfite PCR of the 5’ UTR region of miR-129-2 gene. |
| GAGTTGGGGGATTGTGGAT | Forward unmethylation specific primer for bisulfite PCR of the 5’ UTR region of miR-129-2 gene. |
| AATATACCAACTTCTTCAATTCACCA | Reverse unmethylation specific primer for bisulfite PCR of the 5’ UTR region of miR-129-2 gene. |
| CCGCTCGAGCTGCTCCATTCTCCAGTCTGAACAG | Forward primer for amplify the 3'-UTR of VCP gene containing the putative miR-129-binding sites |
| CGGGATCCGGCTGTTCCCAGGAAAAGAAGCA | Reverse primer for amplify the 3'-UTR of VCP gene containing the putative miR-129-binding sites or deletions of miR-129-binding sites |
| GTTTCTGTTGTACAAAACAAAAGCGATAAAATAAAAG | Forward primer for amplify the 3'-UTR of VCP gene containing the deletion of the first or the two of putative miR-129-binding sites |
| ATCGCTTTTGTTTTGTACAACAGAAACCCCCTGTCC | Reverse primer for amplify the 3'-UTR of VCP gene containing the deletion of the first or the two of putative miR-129-binding sites |
| CAGTTTCTAAACATGACATGTTGTAAAAGGACAATAAAC | Forward primer for amplify the 3'-UTR of VCP gene containing the deletion of the second putative miR-129-binding sites |
| GTCCTTTTACAACATGTCATGTTTAGAAACTGCTTGTG | Reverse primer for amplify the 3'-UTR of VCP gene containing the deletion of the second putative miR-129-binding sites |
| GAACAGGCCAAGACTGAAGC | Forward primer for *CDC25c* RT-PCR |
| GCCCCTGGTTAGAATCTTCC | Reverse primer for *CDC25c* RT-PCR |
| TTTCTTTCGCGCTCTAGCCA | Forward primer for *CDK1* RT-PCR |
| CAATCGGGTAGCCCGTAGAC | Reverse primer for *CDK1* RT-PCR |
| GCCAATGGGAAGGGAGTG | Forward primer for *CCNB1* RT-PCR |
| ACCCAGCAGAAACCAACAGC | Reverse primer for *CCNB1* RT-PCR |
| TGCCCAAGCTCTACCTTCC | Forward primer for *p21* RT-PCR |
| ACAGGTCCACATGGTCTTCC | Reverse primer for *p21* RT-PCR |
| TGAACAATGGGCCTCGTCTG | Forward primer for *Wee1* RT-PCR |
| CTATGGCTCGGGAGTGTCAG | Reverse primer for *Wee1* RT-PCR |
| AAGACCCAGGTCCAGATGAAG | Forward primer for *p53* RT-PCR |
| AGAATGCAAGAAGCCCAGAC | Reverse primer for *p53* RT-PCR |
| GAAGGTCGGAGTCAACGGAT | Forward primer for *GAPDH* RT-PCR |
| CTGGAAGATGGTGATGGGATT | Reverse primer for *GAPDH* RT-PCR |
| ATCCGTGAATCCATCGAGAG | Forward primer for *VCP* RT-PCR |
| GGAATCTGAAGCTGCCAAAG | Reverse primer for *VCP* RT-PCR |

**Supplementary Table 2.** The programs of TD-PCR

| **Steps** | **Cycle conditions** |
| --- | --- |
| Pre-denature | 95°C for 10 min, one cycle |
| TD program 1 | 30 s at 95°C, 30 s at 60°C, 30 s at 72°C, two cycle |
| TD program 2 | 30 s at 95°C, 30 s at 59°C, 30 s at 72°C, two cycle |
| TD program 3 | 30 s at 95°C, 30 s at 58°C, 30 s at 72°C, two cycle |
| TD program 4 | 30 s at 95°C, 30 s at 57°C, 30 s at 72°C, two cycle |
| TD program 5 | 30 s at 95°C, 30 s at 56°C, 30 s at 72°C, two cycle |
| TD program 6 | 30 s at 95°C, 30 s at 55°C, 30 s at 72°C, two cycle |
| TD program 7 | 30 s at 95°C, 30 s at 54°C, 30 s at 72°C, two cycle |
| TD program 8 | 30 s at 95°C, 30 s at 53°C, 30 s at 72°C, two cycle |
| TD program 9 | 30 s at 95°C, 30 s at 52°C, 30 s at 72°C, two cycle |
| TD program 10 | 30 s at 95°C, 30 s at 51°C, 30 s at 72°C, two cycle |
| Main cycle | 30 s at 95°C, 30 s at 50°C, 30 s at 72°C, 15 cycles |
| Final extension | 72°C for 10 min, one cycle |
